# The ‘nuts and bolts’ of including service users and carers in the recruitment of health and social work students in an English university—An interdisciplinary critique

**DOI:** 10.1111/hex.13137

**Published:** 2020-09-28

**Authors:** Peter Unwin, Joy Rooney

**Affiliations:** ^1^ University of Worcester Worcester UK

**Keywords:** interdisciplinary, selection process, service users and carers, student recruitment, student selection

## Abstract

**Background:**

The literature regarding inclusion of service users and carers (SUACs) in the recruitment processes for future health and social work professionals has primarily presented such inclusion as positive for all. This study is novel in its exploration of the detail of SUACs' involvement and in its reach across a whole university department of health and social care disciplines.

**Objective:**

To examine the detail of ways in which SUACs were actually involved in student selection and whether they have any real influence on recruitment decisions.

**Method:**

This co‐produced study took place in an English university. A qualitative, semi‐structured interviewing approach was undertaken with 12 staff across social work, nursing, occupational therapy, physiotherapy, paramedicine and physician associate disciplines. Thematic analysis was employed independently with both researchers agreeing resultant themes.

**Findings:**

A range of recruitment activities which included SUACs were found, evidencing both full and partial involvement in decision making. Nine themes emerged—The quality of SUACs' inclusion; Understanding reasons for including SUACs; SUACs being more knowledgeable than academic staff; SUACs influencing final decisions; The high expectations of candidates by SUACs; SUACs' need for training; Marketization and scepticism; and Logistics and the presumption of ableism.

**Conclusions:**

Transparent protocols are essential if SUACs are to be equitably included in student recruitment processes. A shared model of SUACs' inclusion should be attainable across disciplines, even if the ‘nuts and bolts’ of recruitment processes vary.

**Patient or Public Contribution:**

This work was co‐produced with a SUACs' group from conception and design through to fieldwork and write‐up.

## 1 INTRODUCTION

### 1.1 Background

The involvement of laypeople in the management of professional disciplines came about in the 1980s, partly as a check on professional power but partly also as a way to give a consumer voice to people using the UK health and social care services.^1^ The consumerist model of industry and commerce was seen as being directly transferable to health and social care services where the customer is king and profit the bottom line in a marketized economy.^2^, ^3^ The international picture regarding the inclusion of service users and carers (SUACs) in qualifying health and social care training is rather mixed, despite bodies such as the International Federation of Social Workers and the International Association of Schools of Social Work^4^ setting out global standards for the education and training of the social work profession which call for student recruitment, admission and retention policies that reflect the active involvement of practitioners and service users in relevant processes. The UK bodies such as the Nursing and Midwifery Council and Social Work England are believed to be the only regulators who have made service user involvement mandatory in the recruitment and teaching in their respective disciplines' qualifying courses. Universities across Europe and the rest of the world have begun to incorporate service users into their activities, including recruitment, particularly in the field of social work in the UK and in Eastern European countries.^5^, ^6^ However, user involvement in social work education in the Nordic countries is still limited^7^ while the service user inclusion in the US social work education remains relatively absent.^8^ International higher education establishments have been criticized for principles of ableism, more so since the embracing of performance management models of efficiency.^9^, ^10^ In the UK context, the number of disabled academic staff was self‐reported at 4.9%^11^ whereas 19% of all working‐age adults were stated to have a disability.^12^ The positive role models provided across qualifying health and social care courses by SUACs from recruitment through to involvement in teaching and assessment, might be seen as a challenge to this predominantly ableist culture.

There is a growing evidence base about the involvement of SUACs in the recruitment of students applying for professional health and social care courses in the UK higher education establishments.^13^, ^14^, ^15^ Staff views of the benefits of working with SUACs across their health and social care qualifying courses have largely been reported as positive,^13^, ^15^, ^16^ but no studies have examined the detail of the roles SUACs play in student recruitment, the extent of their involvement in executive decision making and how this might vary across disciplines.

The voice of SUACs in health and social care has brought about some changes within qualifying courses at the UK universities where SUACs are to be found in a number or roles across selection, teaching, quality and research.^17^, ^18^ However, there is no evidence that their presence in such roles has brought about systemic change in student recruitment and only the study by Tanner et al^19^ has attempted to gauge whether the skills, values and knowledge gained by exposure to SUACs from recruitment and in subsequent teaching carries on into qualified practice. Tanner et al^19^ compared two universities, but were unable to disentangle the SUACs' involvement factor among the many personal and organizational factors which impacted on early career experiences. In a climate of public sector cuts and the continuing privatization of services, it is perhaps easier to apply tokenistic models of involvement, rather than models that are more fully meaningful and resource intensive. The lack of prescription in the guidance offered to nursing courses,^20^ and social work courses,^21^ means that qualifying courses might still gain approval, even if the extent of SUACs' involvement is marginal.

The above studies on service user involvement in student recruitment at the UK universities have centred on course, rather than whole department initiatives; the present study helps address that gap in knowledge and is based on research carried out at an English university where a SUACs' group of some 40 individuals work alongside academic staff in selection, teaching, quality forums and research. The group members are trained in recruitment legislation and best practice, and are paid fees plus expenses for all involvement from a dedicated budget. The study, carried out in 2018, was co‐produced by a service user and an academic and interviewed 12 professionals from social work, nursing, occupational therapy, physiotherapy, paramedicine and physician associate courses.

### 1.2 Literature review

The literature regarding lay involvement, and more particularly lay involvement in health and social care selection,^1^ has centred around whether it is tokenistic or meaningful, with debates around a range of issues including representativeness, resourcing and power imbalances.^15^, ^22^, ^23^ The extant literature covers a considerable amount of ground but very few studies consider in any detail the ‘nuts and bolts’ regarding what SUACs' involvement in interviews actually involves.

Since the 1980s, involvement of the public across a range of public organizations in the UK has become commonplace. Then, the Conservative government introduced a series of ‘Citizen's Charters’ designed to provide a check and balance on professionals' spending, but also to give voice to laypeople. Examples include ‘The *Patients' Charter’*,^24^ ‘*A New NHS: Modern, Dependable’* ^25^ and ‘*Modernising Social Service*’,^26^ all of which sought to give new voices to consumers of health and social care. However, a systematic review of 36 international public bodies by Baxter et al^6^ found a lack of clarity about laypeople's roles on public bodies, coupled with an absence of on‐going support and training. A range of models regarding layperson involvement were described in this review, with roles being played by laypeople which ranged from the tokenistic to the fully inclusive, as illustrated below:

- SUACs suggesting questions, but not being involved in social work recruitment.^27^
- A panel of SUACs meeting with nursing applicants prior to interview to ask questions but not becoming involved in the formal interview.^28^
- Separate professional and service user panels which asked specific questions and then met together later to discuss student responses. The service user panel asked about behaviours and character whereas the professional panel asked about skills and knowledge.^29^
- A service user and clinician jointly interviewing applicants.^13^

This systematic review also analysed the involvement of laypeople in the actual scoring/evaluative process of interviews and argued that a transparent strategy should always be available regarding how much weight would be given to stakeholders in the assessment process. Most organizations in this review agreed about the benefits around value base, communication skills and attitudes brought about by lay involvement.^6^

In a wide‐ranging review of social care, the inclusion of SUACs' groups across the UK was viewed as being marginal, rather than core,^30^ despite health and social care organizations being at the forefront of involving laypeople, with nationwide organizations being set up to encourage such participation across the NHS.^31^ The much‐heralded Patient and Public Involvement Initiative (PPI) brought about via the NHS Reorganisation Act 1973 was criticized^32^ as having remained primarily at the tokenistic end of Arnstein's ‘Ladder of citizen participation’ scale.^33^ This scale is a useful theoretical framework by which SUACs' modes of inclusion might be measured and consists of a hierarchical construct of participation, from patronizing models through to tokenistic models (where decisions have already been made) and places models of partnership, delegated authority and citizen control on its top rungs.

However, other research found confusion among some stakeholders regarding the purpose of SUAC involvement—one set of views were that their involvement was to bring new knowledge and insights to the process, whereas other stakeholders viewed the involvement of SUACs as being to help redress the power of professionals, as well as to help the individual SUACs' representatives' own development.^34^ Other studies have been confident in stating the benefits to staff of having SUACs involved in student selection—one study found that decision making across two courses relating to the selection of post‐graduate clinical psychologists was significantly improved when SUACs were involved in the process,^17^ while another study across a range of disciplines^15^ found that working with SUACs acted both as a stimulus to staff practice and a reminder of their professional value base.

The advent of values‐based recruitment in nursing introduced a system whereby candidates are chosen against the core NHS principles of respect and dignity; quality of care; compassion; improving lives; and working together.^20^ This system came about partly as a response to nursing scandals and also as a check on marketized values. The use of ‘mini‐stations’, to assess candidates, with a variety of activities at each station, is a model which has become commonplace in values‐based recruitment of students.^35^ An example of values‐based recruitment from a study of learning disability student nurse recruitment was an exercise designed to enable candidates to see issues from a learning disabled person's perspective.^5^ Five SUACs were present and encouraged to illuminate the scenarios by reference to their own lived experience to bring a sense of reality into the scenarios.

The question arises whether a less than full involvement in any recruitment process militates against the likelihood of challenge to professionals' perspectives. The need for some distance between SUACs and the recruiting organization is perhaps ever more important when universities are having to compete for student numbers, in order to survive financially. There is also the argument that ‘we are all service users and carers’, that is all health and social care professionals have their own lived experiences, hence are already in a position of being able to take a SUACs' perspective. This argument is criticized by other authors whose view is that the experiences of professional staff, who have the benefit of employment and status, cannot equitably be compared with the lives of many service users and carers, whose lives do not share such levels of privilege.^36^ However, particularly in the field of mental health, this rather dichotomous argument perhaps needs to be more fluid.

Resourcing the involvement of laypeople has been acknowledged as a source of difficulty, particularly at times of austerity and its associated public sector cutbacks. A key principle of lay involvement, and certainly of the involvement of SUACs within health and social care, is that they should receive payment for their time and expertise.^37^ Others noted that, in the absence of funding, the involvement of SUACs in social work student recruitment was limited to suggesting questions that might be asked.^25^ However, more researchers stressed the need to properly resource SUACs' involvement, arguing that the power balance in decision making was clearly biased towards the professionals.^28^, ^38^ A system whereby nursing students wrote a piece on values which was assessed by service users using standardized grading was also reported.^39^ Service users, however, were not present at the subsequent interviews where their questions were posed by academics and practitioners. The rationale given for this process is that it enabled the involvement of service users whose personal limitations might have made face‐to‐face interviews difficult.^39^

Some universities have introduced audio‐visual technology into their recruitment models but usage of video recordings in place of service users being present was viewed in one study as a reductionist model.^40^ Others described a system whereby nursing candidates watched video recordings of service users asking questions, which they subsequently answered on audio recording as if the service users were actually present.^41^ Such usage of technology possibly reflected pressures on logistics and resourcing.^3^ At the tokenistic end of the spectrum, in a social work course, the academics held reservations about the decision‐making qualities of service users in student interviews, and accordingly took the executive decision‐making powers themselves.^13^ Other studies address such issues of power inequality in recommending that a clear rationale should be present for the voting/assessment strategy regarding selection criteria and that there should always be a pre‐agreed plan in the event of a significant disagreement.^13^, ^40^

The need to sufficiently train and orientate SUACs in respect of the processes of recruitment was viewed as key to success.^6^, ^38^ However, insisting on conformity with university human resources (HR)' diversity and equality training courses can be viewed as part of an assimilation process which discourages discussion and challenge. Such HR procedures and protocols are essentially designed to assure all concerned, not least student applicants, that theirs would be a competent, fair and equitable recruitment process, referenced against key legal requirements.

The findings of the above studies suggest that there is still some way to go before power imbalances in SUACs' involvement in student recruitment are redressed. The report project reported below was designed to illuminate such issues by studying the ‘nuts and bolts’ of the ways in which SUACs are actually involved in student recruitment.

## 2 METHODOLOGY

### 2.1 Design and setting

The research was co‐produced from conception to write‐up, the university's SUACs' group having raised initial interest in the topic. Members of this group undertake a preparation course for their role in recruitment which takes place over two days, covering key issues of safeguarding, ethics and boundaries, supplemented by the university's core offering of an online equality and diversity training package. A volunteer from the SUACs' group and an academic staff member subsequently led the research, using the SUACs' group as a reference point as the study progressed. Both researchers have many years of research experience and had previously worked together on the successful co‐production of research. Closed and open interview questions were drawn up asking technical questions about working with SUACs and about whether staff felt such involvement contributed to the process, what advantages and barriers they might identify and whether there were any areas in which such inclusion could be made more effective.

All teaching staff (n. 28) involved in the recruitment of pre‐registration health and social work departments in the university were contacted by e‐mail explaining that volunteers were needed to take part in semi‐structured interviews exploring the ways in which SUACs were deployed across the disciplines. Uptake was slow, many staff citing pressures of work as prohibitive factors to giving up an hour for the research. A reminder e‐mail was sent out after only five staff initially expressed interest, this second e‐mail producing a further seven participants. Ethical approval was gained from the university, and the standard set of information, consent forms and details of withdrawal and anonymity/confidentiality were given to participants.

### 2.2 Staff perspectives

Arranging the interviews proved difficult due to timetabling pressures and several ‘no‐shows’ led to rearrangements being made. There is always a tension when conducting ‘insider’ research,^42^ the researchers being careful to emphasize they were seeking critique, even though they were part of the service being studied. The data that did emerge seemed candid and there were several areas of negative criticism/areas for improvement which suggested that respondents were not overly inhibited.

### 2.3 Analysis

Interviews were audio‐recorded and transcribed before being independently thematically analysed^43^ by both researchers. Once saturation point was reached in the independent drawing up of two sets of initial codings, these were progressively distilled into the nine themes reported below.

### 2.4 Proviso

The interview data below are not ascribed to individual professionals because the small number of participants from each discipline would have made them easily identifiable. The findings and discussion below are presented generically, partly to avoid identification of the specific participants, but also because the value bases of health and social care courses are very similar in nature, even if the techniques and the ‘nuts and bolts’ of their interview processes are different.

## 3 FINDINGS

### 3.1 Grid of activities and SUACs' role

The grid of interview activities indicates which professions make the most varied use of SUACs in interviews (Table 1). Individual disciplines used a variety of exercises and interview systems to recruit students, the extent and nature of this SUACs' collaboration varying across disciplines.

**TABLE 1:** Grid of activities and SUACs' role

Course	Nos	View UCAS form	Activity 1	Activity 2	Activity 3	Activity 4	Activity 5	Final decision inclusion
SW	20	No	Written exercise	Interview A/P + SUAC	Role play SUAC + A/P or SUAC × 2	Group discussion SUAC × 2		No
Nurs.	30	Yes	Written exercise	Interview A/P	Numeracy test	Role play SUAC	Observed group task A/P + SUAC	No
OT/PT	20	No	Group task 1 A/P × 2 + SUAC	Group task 2 A/P × 2 + SUAC	Interview A/P or SUAC			Yes
Param.	30	No	Group task (6 groups) A/P + SUAC × 2 in a pair	Interview (if pass group task) A + P				No
PA	12	No	8 mini‐stations A/Ps + SUAC	Interview A + P				No

Abbreviations: A, academics; Nos, numbers of students invited to interview days; OT, occupational therapy; P, practitioners; PA, physician associates; Param, paramedics; PT, physiotherapy; SW, social work; Nurs, nursing (mental health, adult, children's); SUAC, service user and carer; UCAS, university application form.

Nursing course recruitment events included SUACs in five activities; social work in four activities; occupational therapy/physiotherapy in three activities with physician associates and paramedics using them in two settings. Group activities ranged from creating a profile of a health practitioner's qualities and the building of a tower, through to discussions on case studies. Staff teams had all constructed their own systems and these were reported as constantly changing and being adapted, in some cases due to logistical pressures of staff time and rooming issues. Only adult nursing gave SUACs sight of each student's application form.

### 3.2 Theme 1. The quality of involvement of SUACs

The quality of SUACs' contribution to the recruitment process was widely acknowledged across all disciplines:

I can see value. Personally, I can see value from a non‐academic perspective being brought into a selection process. (Staff 1)

I think they're always consistent. I think the standard of the service users that they're really well briefed, they know what to do. (Staff 10)

To me it's invaluable, it really is to get a different perspective on a candidate, to give a good picture of that person from different viewpoints, I think is invaluable. (Staff 11)

I think it's a ‘real eye’ perspective. They bring personal experience from skills that they have in life, and also from the conditions that they may live with. So we've had all sorts. We've had people who are on oxygen, people who are partially blind who bring their dogs in. So, it's about getting quality of the right candidates for the future of our course really. (Staff 12)

### 3.3 Theme 2. Understanding reasons for including SUACs

Most staff clearly understood why they included SUACs, valued their distinct contributions, and were able to articulate their contribution:

It's a co‐production between the university and all stakeholders to get the best candidates for the next generation. (Staff 2)

Service users and carers were involved as partners, were involved in the setting up, the design of the new process, involved in the group work assessment and the role play, so involved in assessing that on an equal level as academics, and have been involved in reviewing that on an annual basis. (Staff 7)

### 3.4 Theme 3. SUACs being more knowledgeable about processes than academic staff

The three staff quotes below indicate that service users informally manage the process through their familiarization with a system they are likely to have been party to designing:

Some of them in my experience know and understand the process better than some of the academics who get involved because they're involved so much and across the institute they're involved so much. So certainly sometimes I've turned up and the service users have been reminding me where I have to be and what I have to do, so that's helpful. (Staff 5)

So, they were able to help and also guide and support me through the process of it. And when we were summing it up and evaluating at the end, shared their thoughts, their opinions on things that I may have missed. (Staff 8)

One of the biggest things that I've gained, is working with a service user who had more experience than I did in doing the roleplay, and listening to it from her perspective and engaging at a professional level, so getting the feedback from her and she made some suggestions, and I thought ‘Oh that's really good, yeah I'm going to use that, yeah I'll use that’. (Staff 8)

### 3.5 Theme 4. SUACs influencing final decision making

Examples were given where the influence of a SUAC view was critical to the final decision:

There's been a patient interaction station that has in essence been virtually run by the service users; marked by them. (Staff 6)

There was a candidate I really wanted. I thought she was fantastic, she was just assertive I thought in quite a good way. I thought she would be really valuable to the course, academically inclined, and the SUAC member in the group said ‘This person, I've seen her talk over other people, I find her behaviour quite offensive and I don't think she has the values to be on the course’. And in the discussion we decided not to take her because of that perspective. (Staff 2)

It has happened, that we haven't offered a place to an applicant because the service user member of the panel has said ‘I really don't think this person is suitable, you know, they blanked me, they didn't kind of acknowledge that I was there as a service user’ or something like that. (Staff 7)

### 3.6 Theme 5. The high expectations held by SUACs about candidates

The experience of academics regarding the stage of potential/development they expect candidates to have reached by interview stage sometimes came up as an area of disagreement between SUACs and academic staff:

I'm not saying that we always need to agree, but there have been instances where I think maybe the service user has expected too much from a candidate, and has unrealistic expectations and maybe sometimes fails to see potential in people and expects the full package to come. (Staff 4)

We've had incidences, with the group work for example where a clinician or a SUAC member has got a bit too involved and actually we want them to leave them to it and just observe. (Staff 3)

I think there may be a challenge where, particularly with the new SUAC member, that the expectations are too high. So it's having that dialogue about what is the starting point. (Staff 8)

### 3.7 Theme 6. SUACs need for training while maintaining their uniqueness

The wider literature regarding SUACs' inclusion in higher education recognizes that their unique, lived perspectives of services are what distinguish their contributions,^15^, ^22^ while recognizing the challenges in achieving ethical working relationships with academic staff, as discussed in the following quotes:

Service users and carers aren't robots. They're not along to do what we want them to do; they're here to bring their own perspective. And so sometimes that can be challenging to work with, especially if somebody's got a lot to say about a particular applicant or their own experience. (Staff 7)

The above quote from Staff 7 about SUACs not being present in recruitment scenarios for the benefit of the institution is qualified by the following staff quote:

I think the potential disadvantages could be that they are not sufficiently prepared, involved with or engaged in the process of training beforehand. So there is a training need and a support need to ensure that they understand what the university needs out of the selection process, as well as engaging them to find out what would be useful as well. (Staff 8)

### 3.8 Theme 7. Equal inclusion of all participants

Some staff welcomed the opportunity to discuss the inclusion debate with the researchers and were clear that all individuals involved in the recruitment of students should have meaningful roles, informed by guidelines regarding expectations:

It doesn't matter whether it's a staff member, a SUAC or clinician; we usually improvise groups so everybody has a group. We tend to swap groups. And then for the one‐to‐one questions the SUAC member will have a group of students, the clinician will have a group of students… So hopefully it's not tokenistic. (Staff 3)

What we're looking at when we've revised our processes this year is actually having a bit of a check list of what we expect from SUAC members and clinicians because they're both coming in. So, it'd be the same list for clinicians and SUAC members about what we're expecting them to do. (Staff 3)

The nature and extent of SUACs' involvement differed significantly across a variety of group tasks, observation tasks and standard interviewing tasks. In one area (occupational therapy/physiotherapy), SUACs were fully involved as equals in decision making, selecting the final candidates and took part in feedback regarding whether a candidate was successful overall on their selection day. For other courses, SUACs were not involved in scoring all activities and did not have equality of opportunity to have an executive say on successful candidates.

### 3.9 Theme 8. Marketization and scepticism

A small number of staff expressed scepticism about the real value that SUACs brought to student recruitment, viewing the demands of the institution as paramount. Staff members spoke about how the marketization of many health and social care courses had put pressure on staff regarding achieving full recruitment numbers and having to ‘sell’ their programmes. Under such imperatives, it is perhaps not surprising that SUACs' views might be overridden or SUACs with more complex needs might prove too challenging logistically to include in a fast‐paced system:

Very occasionally some service users are very harsh in their judgement of the students. Just occasionally we have found that, and it's always interesting and it's always a really good discussion point, is that the team will be happy to offer a place and the service user has been really unhappy with the student. (Staff 6)

We changed the structure of the selection day because we wanted just to streamline the process. It was just purely a marketing thing, just to attract people because we had been told our selection was a little bit too tough in terms of how many exercises we had… it was not related to reducing or changing service users' involvement. (Staff 9)

It's very difficult when you have processes that are very fast paced, that are looking at recruiting any numbers. (Staff 2)

### 3.10 Theme 9. Logistics and the presumption of ableism

The logistical complexities of including SUACs with complex needs was commented on as being a ‘downside’ in the increasingly pressured higher education environment:

There's the technical downside to some of our service users who have got complex needs, which requires us to have a lot of support strategies in place to allow them to help with the day. (Staff 6)

Staff 2's comments below about the inherent ableism among professionals raise a significant challenge to current practices regarding SUACs involvement across student recruitment:

Ableism is inherent in the way people plan things. And it's not even understood because people see themselves as healthcare professionals or professionals in academia and see themselves as quite liberal and see themselves as someone who would never discriminate, but there's inherent decisions that automatically exclude. (Staff 2)

## 4 DISCUSSION

This study is believed to represent the first across a whole university department and involved a wide range of professions (social work, nursing, occupational therapy, physiotherapy, paramedicine and physician associates), giving their views regarding the best ways to work with SUACs in student recruitment.

Also, for the first time, SUACs were reported as knowing the recruitment system better than the academic staff. However, this novel finding of respect for SUACS' knowledge and insights into candidate potential was not extended to their playing a part in the final decision making across all disciplines, which concurs with the findings of previous studies.^28^, ^30^, ^34^

While most staff believed that SUACs were of high quality, there were a small number of sceptical views apparent in this research, particularly against a background of marketization and the managerial imperatives to recruit to maximum numbers across courses. These views are resonant with previous findings.^3^, ^15^

The nature and extent of SUACs' involvement differed significantly across a variety of group tasks, observation tasks and standard interviewing tasks. In one area (occupational therapy/physiotherapy), SUACs were fully involved as equals in decision making, selecting the final candidates and taking part in feedback regarding whether a candidate was successful overall on their selection day. For other courses, SUACs were not involved in scoring all activities and did not have equality of opportunity to have an executive say on successful candidates. This finding echoed earlier work which reported academics having reservations about such holistic involvement and choosing to make the executive decisions themselves.^13^ This disparity in involvement was also evident in the range of models previously identified,^21^ although most staff did not recognize that some practices, such as a partial attendance in the process, and not being involved in the final decision, might be seen as tokenistic. Clear rationales for decision making did not always appear to be the case, although the findings above indicate healthy debate in this area. The necessity of agreeing clear rationales in case of disagreements prior to selection processes taking place was highlighted, supporting previous research findings.^13^, ^40^

This study was small, and bias may have existed in reasons why some staff put themselves forward, while others did not. Issues of potential bias may have included peers not wishing to reveal any poor practice, and the presence of a SUACs' representative in the research team may also have inhibited take‐up. While this SUAC member was not involved in recruitment, staff possibly may have felt they could not be critical of SUACs' involvement in their presence.

The logistics of time and environment were seen as problematic by some staff, especially given pressure to fill courses, such views again having resonance with previous authors.^3^, ^15^ However, each set of professionals were happy with their own selection process involving SUACs and all kept their systems under active review. One staff member raised the challenging point that ableism tended to be dominant in staff‐SUACs' discourses, more so at times of economic restraint, and the adoption of business models by universities.^3^, ^10^, ^15^

## 5 CONCLUSION AND RECOMMENDATIONS

This study differed from previous studies of SUACs' involvement in student recruitment in that it covered a whole academic department and examined the ‘nuts and bolts’ of what actually happens in specific recruitment processes. Despite the reservations that some staff held about tokenistic tendencies, the findings largely complement previous findings that present SUACs' inclusion in student recruitment as making a positive contribution. The need for preparation, orientation and transparent protocols is essential for staff, practitioners and SUACs alike if shared recruitment processes are to be productive.

Recommendations are that different disciplines might exchange ideas and experiences on an annual basis about what is working well regarding SUACs' involvement in student recruitment activities. This would facilitate the sharing of the different ideas and experiences such as those which emerged in the above research. A shared model of the purpose of SUACs' involvement in student recruitment could bring richness and rigour across all health and social care disciplines, even if the ‘nuts and bolts’ of each discipline's activities are different.

## CONFLICT OF INTEREST

The authors declared no potential conflicts of interest with respect to the research, authorship and/or publication of this article.

## Data Availability

The data that support the findings of this study are not publicly available due to privacy and ethical restrictions given risk of identification in a one site study.
